# Determinants of Use of Insecticide Treated Nets for the Prevention of Malaria in Pregnancy: Jinja, Uganda

**DOI:** 10.1371/journal.pone.0039712

**Published:** 2012-06-22

**Authors:** Laura R. Sangaré, Noel S. Weiss, Paula E. Brentlinger, Barbra A. Richardson, Sarah G. Staedke, Mpungu S. Kiwuwa, Andy Stergachis

**Affiliations:** 1 Department of Global Health, University of Washington, Seattle, Washington, United States of America; 2 Department of Epidemiology, University of Washington, Seattle, Washington, United States of America; 3 Department of Biostatistics, University of Washington, Seattle, Washington, United States of America; 4 School of Medicine, London School of Hygiene and Tropical Medicine, Makerere University, Mulago Hospital Complex, Malaria House, Kampala, Uganda; 5 Clinical Epidemiology, School of Medicine, Makerere University, Kampala, Uganda; 6 Global Medicine Program, Departments of Epidemiology and Global Health, University of Washington, Seattle, Washington, United States of America; Chancellor College, University of Malawi, Malawi

## Abstract

**Background:**

One established means of preventing the adverse consequences of malaria during pregnancy is sleeping under an insecticide treated net (ITN) throughout pregnancy. Despite increased access to this intervention over time, consistent ITN use during pregnancy remains relatively uncommon in sub-Saharan Africa.

**Methodology/Principal Findings:**

We sought to identify determinants of ITN use during pregnancy. Utilizing a population-based random sample, we interviewed 500 women living in Jinja, Uganda, who had been pregnant in the past year. ITN ownership at the start of pregnancy was reported by 359 women (72%) and 28 women (20%) acquired an ITN after the first trimester of pregnancy. Among 387 ITN owners, 73% reported either always sleeping under the ITN during all trimesters of pregnancy, or after acquiring their net. Owning more than 1 net was slightly associated with always sleeping under an ITN during pregnancy (RR: 1.13; 95% CI: 1.00, 1.28). Women who always slept under an ITN during pregnancy were more likely to be influenced by an advertisement on the radio/poster than being given an ITN free of charge (RR: 1.48; 95% CI: 1.24, 1.76). No differences were found between other socio-demographic factors, pregnancy history, ANC use or socio-cultural factors.

**Conclusions/Significance:**

While self-reported ITN ownership and use was common throughout pregnancy, we were unable to pinpoint why a sizable fraction of Ugandan women did not always adhere to recommendations for use of an ITN during pregnancy. More data are needed on the capacity of individual households to support the installation of ITNs which may provide insight into interventions targeted at improving the convenience and adherence of daily ITN use.

## Introduction

In areas with stable malaria transmission, malaria parasitaemia is commonly asymptomatic during pregnancy. However, the adverse consequences of malaria infection can be substantial among both symptomatic and asymptomatic women [1]–[4]. Effective prevention strategies and case management of malarial illness are therefore the foundation of malaria control during pregnancy. A meta-analysis on the effectiveness of ITNs in preventing adverse maternal and fetal outcomes in Africa among pregnant women living in areas with endemic malaria concluded that the use of ITNs during pregnancy reduced placental malaria by 23%, reduced fetal loss by 32% and improved birth weight by 33 grams [5].

ITNs are distributed free of charge to all age groups in 38 countries within the African region and a majority of the remaining countries have mechanisms to sell ITNs at subsidized prices [6]. While mass, free ITN distribution campaigns and subsidized voucher programs for ITNs targeting pregnant women and children <5 years have been successful at increasing the proportion of households that own and use an ITN [7]–[11], ITN use among currently pregnant women in many intervention areas remains low [8], [10]. A recent synthesis of coverage data estimates that 23 million pregnancies in sub-Saharan Africa were unprotected by ITNs in 2007 [12]. The 2009 Malaria Indicator Survey in Uganda reports 53.7% of pregnant women slept under any mosquito net the night before the interview (an increase from 24 percent in 2006), and 43.7% slept under an ITN (an increase from 10 percent in 2006) [13], [14]. Although, among pregnant women who owned an ITN, the prevalence of usage the night before the survey increased to 77% [13].

While the gap between the prevalence of ITN ownership and usage has been reported [15]–[17], less is known about the individual-level factors associated with bed net use during pregnancy among households that own a net. Previously reported demographic associations have been inconsistently described across studies [18]–[22]. A small number of qualitative studies have identified recurring themes among women related to bed net usage during pregnancy, including limited knowledge of malaria in pregnancy, doubts about ITN effectiveness, and safety of insecticides during pregnancy, as well as barriers to bed net use including cost, lack of hardware to hang bed nets and high ambient temperatures [23]–[26].

Considering the ongoing gap between ITN ownership and usage commonly observed in Africa and the recent changes in national policy to distribute ITNs free of charge, an investigation of factors influencing a women's decision use an ITN during pregnancy is warranted. We utilized a population-based sample to provide information to support the Ugandan National Malaria Control Program with their policy objective to have all pregnant women sleep under an ITN throughout pregnancy. Specifically, we sought to identify determinants of use of ITNs during pregnancy in Jinja, Uganda.

## Methods

### Selection of study participants

Home-based interviews were conducted between November, 2008 and January, 2009 from a population-based simple random sample of 500 female residents of Kibibi and Namizi parishes in Budondo-sub county of Jinja District, Uganda, to ascertain ownership, acquisition and use of ITNs and antimalarial use during pregnancy, as well as possible factors associated with these behaviors. To be eligible, women needed to be between the ages of 15 and 49 years, have been pregnant within the past 12 months, and the pregnancy must have lasted until at least the third trimester regardless of pregnancy outcome. Details of the study population and procedures have been previously described [27].

### Study site

A population-based sample was selected from a census completed in November 2008 by the Ugandan Malaria Surveillance Project. The study site included 16 villages within Kibibi and Namizi parishes constituting a population of 21,681 residents. There were 4,654 women aged 15–49 years, 867 of whom reported having been pregnant in the previous 12 months.

### Data collection

Information was collected using a pre-tested interviewer-administered structured questionnaire. The questionnaire was based on a conceptual framework adapted from Ribera et al [28] and ascertained information on socio-demographic characteristics, socio-cultural factors, obstetric history, knowledge and attitude of malaria, antenatal clinic use, and ITN ownership and use. Wherever possible, questions were taken from those used in large international surveys, including the Uganda Demographic and Health Survey, and the Malaria Indicator Survey [14], [29]. The questionnaire was translated from English to Lusoga and back to English.

Each woman recounted episodes of self-reported malaria, any antimalarial use during pregnancy, and antenatal care (ANC) visits using a pregnancy history calendar. To assess frequency of usage of bed nets, we asked women to tell us how often (always, sometimes, rarely, or never) they slept under a mosquito net during months 1–3, months 4–6, and months 7–9 of the index pregnancy. For each time period, we showed women the corresponding months on the calendar and then asked for their response. To improve recall, the calendars contained national holidays and other culturally significant dates.

### Definitions

In this community, two types of pretreated nets were available, either conventional ITNs marketed to have an effective level of insecticide for up to 1 year, or long-lasting ITNs (LLINs) which do not need to be retreated for up to 5 years. Households in the study area may have acquired a net free of charge through a mass distribution program targeting pregnant women and children less than 5 years, through a community-based subsidized net distribution program, or they could have purchased a net through a local vendor. Free or subsidized ITN distribution was not available through the ANC clinics in the study area. To help identify the type of net women were using, we showed them photographs of the nets inside packages most commonly available in their communities. ITN usage was ascertained separately for each trimester of pregnancy. Consistent ITN use was defined by the women responding she always slept under an ITN for each trimester that her household owned an ITN. For each trimester, she was shown the corresponding months on the calendar.

### Statistical analysis

Analyses were performed using Stata version 11.0 (College Station, Texas, USA). Primary analyses identified correlates of self-reported consistent ITN use throughout all trimesters of pregnancy (as defined above), among women who had been pregnant within the past 12 months, which included: age, marital status, education, religion, parish, pregnancy history, knowledge of malaria, and socio-cultural factors. A composite variable was generated to summarize a woman's knowledge of malaria based on 7 questions regarding malaria transmission during pregnancy, consequences of infection, and prevention. The maximum score possible was 9.5. Responses were scored, and then the distribution was dichotomized at the mean value. Principal components analysis was used to calculate the household wealth index, a standardized composite measure combining the cumulative living standard of a household and is based on a household's ownership of selected assets, such as televisions and bicycles, materials used for housing construction, and types of water access and sanitation facilities [30]. The association between these factors and use of an ITN during pregnancy was evaluated using relative risk regression [31], [32]. Multivariate models were developed to assess the independent effects of the various correlates. Covariates thought to be associated with both the outcome and exposure of interest were identified a priori. No differences were found between unadjusted and adjusted risk estimates, indicating that confounding by the measured variables is unlikely to be biasing the results. Therefore, adjusted estimates are not presented.

### Ethical approval

The study was approved by the Research and Ethics Committee Faculty of Medicine Makerere University (MUREC), the Uganda National Council for Science and Technology, and the University of Washington Institutional Review Board. All participants provided written informed consent. Women less than 18 years of age who have been pregnant are considered emancipated minors and therefore can independently provide informed consent to participate in research [33].

## Results

### Characteristics of the study population

A total of 629 households were visited between November 2008 and January 2009 to identify 500 eligible women, and all eligible women participated in the survey. Details of eligibility, participation and characteristics of study subjects have been previously published [27], [34]. The mean age of the participants was 26 years (range 15–49 years), and the majority of the women were married (90%). The primary religions were Muslim (39%), Protestant (32%), Catholic (20%), or other Christian (9%). Most of the women were living in rural villages (74%).

### Perception of malaria risk in study participants

The risk perception of malaria in these communities is high, with 70% of women responding they were specifically worried about getting sick with malaria during their most recent pregnancy. The primary fears with having malaria in pregnancy were miscarriage/stillbirth (50%), maternal death (16%), and the baby being adversely affected (12%). Self-reported malaria during the most recent pregnancy was very common (67%), and 37% of women reported more than 1 malaria episode. Fifty-eight percent of women had a high score on the knowledge of malaria assessment.

### ITN ownership and acquisition during pregnancy

Seventy-two percent of women reported that they owned an ITN at the start of their most recent pregnancy (n = 359). Among the 141 women who did not own an ITN at the start of the index pregnancy, 28 women acquired an ITN during pregnancy. Women reported acquiring an ITN either during the 2^nd^ (57%) or the 3^rd^ (43%) trimester of pregnancy. All of the women who acquired the net during pregnancy reported always sleeping under it for the remainder of their pregnancy.

### ITN usage during pregnancy

Among ITN owners (n = 387), 73% of women reported either always sleeping under the net during all trimesters of pregnancy, or always sleeping under the net after they acquired one during pregnancy. The primary reason for not always sleeping under the net was the heat (49%). Other reasons are listed in Table 1. Women of Muslim religion were less likely to always use a net during pregnancy compared to those of Christian religions (RR: 0.83; 95% CI: 0.72, 0.95) (Table 2). Owning more than 1 net was associated with a slightly increased likelihood of always sleeping under a net during pregnancy (RR: 1.13; 95% CI: 1.00, 1.28). Women in the wealthiest households were less likely to always use a net during pregnancy compared to women living in the poorest households (RR: 0.77; 95% CI: 0.60, 0.97). Participants were read a list of items and then asked to choose which item is the single most important influence over deciding to sleep under an ITN during pregnancy. Women who always slept under an ITN during pregnancy were more likely to be influenced by an advertisement on the radio/poster than being given an ITN free of charge (RR: 1.48; 95% CI: 1.24, 1.76). Paradoxically, women who always used their net during pregnancy were also slightly more likely to believe that sleeping under an ITN might be dangerous during pregnancy (RR: 1.23; 95% CI: 1.06, 1.42). No differences were found between other socio-demographic factors, pregnancy history, ANC use or socio-cultural factors.

**Table 1:** Descriptive bed net usage information among women who did not always user an ITN.

Reasons for not always using an ITN; n (%)	Did not always use ITN n = 106
Too hot	52 (49.1)
Feel as though I'm suffocating	13 (12.3)
Other*	9 (8.5)
Chemical smell	8 (7.5)
Inconvenience	7 (6.6)
Not necessary	7 (6.6)
Nets are for the kids, do not have extra for myself	6 (5.7)
Not enough space	4 (3.8)

* Other Reasons: Torn/damaged (n = 3); Needed retreatment (n = 1); Needed washing (n = 3); Irregular mosquitos (n = 1); Made me sick (n = 1).

**Table 2:** Predictors of self-reported ITN use during pregnancy among women in Jinja district, Uganda who owned an ITN.

Characteristic	Always ITN use n = 281	Did not always use ITN n = 106	RR (95% CI)
Socio-demographic factors			
Age (years); n (%)			
≤18 years	20 (60.6)	13 (39.4)	0.82 (0.61, 1.10)
19 – 24 years	115 (73.7)	41 (26.3)	Reference
25 – 34 years	108 (72.5)	41 (27.5)	0.98 (0.85, 1.12)
≥35 years	38 (77.6)	11 (22.4)	1.05 (0.88, 1.26)
Marital status; n (%)			
Single	19 (65.5)	10 (34.5)	0.89 (0.68,1.17)
Married	262 (73.2)	96 (26.8)	Reference
Education; n (%)			
None	13 (65.0)	7 (35.0)	0.87 (0.63, 1.21)
Primary	204 (74.7)	69 (25.3)	Reference
Secondary/Postsecondary	64 (68.1)	30 (31.9)	0.91 (0.78, 1.06)
Religion; n (%)			
Christian	187 (77.6)	54 (22.4)	Reference
Muslim	94 (64.4)	52 (35.6)	0.83 (0.72, 0.95)
Parish; n (%)			
Kibibi	129 (70.1)	55 (29.9)	Reference
Namizi	152 (74.9)	51 (25.1)	1.07 (0.94, 1.21)
Village type; n (%)			
Rural	217 (74.6)	74 (25.4)	1.12 (0.96, 1.31)
Peri-Urban	64 (66.7)	32 (33.3)	Reference
Household wealth index; n (%)			
1 (Most poor)	53 (79.1)	14 (20.9)	Reference
2	58 (75.3)	19 (24.7)	0.95 (0.80 1.14)
3	68 (78.2)	19 (21.8)	0.99 (0.84, 1.17)
4	60 (70.6)	25 (29.4)	0.89 (0.74, 1.07)
5 (Least poor)	37 (60.7)	24 (39.3)	0.77 (0.60, 0.97)
Use of ANC			
# ANC visits; mean (sd)	3.2 (1.2)	3.2 (1.2)	0.99 (0.94, 1.04)
ANC initiation; n (%)			
1^st^ trimester	53 (19.1)	23 (22.1)	0.95 (0.81, 1.12)
2^nd^ trimester	183 (65.8)	67 (64.4)	Reference
3^rd^ trimester	42 (15.1)	14 (13.5)	1.02 (0.87, 1.21)
Pregnancy history			
Number of births; n (%)			
1 birth	56 (70.9)	23 (29.1)	0.98 (0.83, 1.15)
>1 birth	225 (73.0)	83 (27.0)	Reference
Prior miscarriage; n (%)			
Yes	48 (68.6)	22 (31.4)	0.93 (0.79, 1.11)
No	233 (73.5)	84 (26.5)	Reference
Prior stillbirth; n (%)			
Yes	16 (84.2)	3 (15.8)	1.17 (0.95, 1.43)
No	265 (72.0)	103 (28.0)	Reference
Knowledge of malaria			
Knowledge of malaria score; mean (sd)	6.3 (1.3)	6.4 (1.4)	0.98 (0.94, 1.02)*
Socio-cultural factors			
Who decides if an ITN is used during pregnancy? n (%)			
Respondent	51 (68.0)	24 (32.0)	0.94 (0.71, 1.23)
Husband/partner	21 (72.4)	8 (27.6)	Reference
Respondent and husband jointly	165 (75.3)	54 (24.7)	1.04 (0.82, 1.32)
Someone else	44 (68.8)	20 (31.2)	0.95 (0.72, 1.25)
Bed net factors			
# Bed nets owned during pregnancy; n (%)			
1 bed net	130 (46.3)	61 (57.6)	Reference
≥2 bed net	151 (53.7)	45 (42.4)	1.13 (1.00, 1.28)
Belief ITN is safe during pregnancy; n (%)			
Yes	253 (71.3)	102 (28.7)	Reference
No	28 (87.5)	4 (12.5)	1.23 (1.06, 1.42)
Most important influence to use ITN; n (%)			
Given free ITN	60 (64.5)	33 (35.5)	Reference
Told by a doctor or nurse	137 (72.9)	51 (27.1)	1.13 (0.95, 1.34)
Ad on radio or poster	22 (95.6)	1 (4.4)	1.48 (1.24, 1.76)
Hearing from other pregnant women	27 (71.0)	11 (29.0)	1.10 (0.86, 1.42)
Having an extra net for kids	35 (77.8)	10 (22.2)	1.21 (0.97, 1.50)

* Comparison based on each one-unit increase in knowledge of malaria score.

## Discussion

In this population-based sample of 500 recently pregnant women in Jinja, Uganda, self-reported ITN ownership was common, as was self-reported ITN usage throughout pregnancy, with 73% of women who owned an ITN reporting that they always used it throughout pregnancy. Only 11% of ITN owners said they never used the net anytime during pregnancy. Among all women sampled, 56% were adherent throughout pregnancy. Reasons for ITN adherence were not well predicted by the characteristics ascertained in this study. Among ITN owners, women of Muslim religion were slightly less likely to always sleep under an ITN and women who owned two or more nets were slightly more likely to consistently use an ITN during pregnancy.

The prevalence of ITN usage among women in Jinja district appears to be similar to that of other pregnant women in Uganda. According to the 2009 Malaria Indicator Survey, among pregnant women in households who own an ITN, 77% slept under an ITN the night before the survey [13]. Our results are also similar to that of UMSP census data of currently pregnant women aged 15–49 years in Jinja District. Bed net usage the night before the census survey was 55% among all currently pregnant women, and 76% among those who own a bed net. Whereas, among the general population of women of reproductive age, only 44% used a net the night before the census survey, and 66% of bed net owners used a net the night before the survey. While ITN coverage and usage in Jinja is higher than that of other settings, they have yet to met the goal to achieve universal coverage of malaria interventions by 2010 set by the UN Secretary General in 2008 [6].

While the prevalence of ITN ownership was relatively high in the study population, 23% of women did not own an ITN during the index pregnancy, highlighting the importance of improving access to ITNs. To scale-up and sustain ITN coverage, “catch-up” and “keep-up” strategies modeled after childhood vaccination programs are gaining popularity [9]. Mass distribution of free ITNs (catch-up) followed by routinely providing ITNs or subsidized vouchers for ITNs to pregnant women and/or children through public health clinics or commercial outlets (keep-up) has dramatically increased coverage and usage in the intervention areas [9]. Likewise, free mass distribution of ITNs alone or subsidized voucher programs alone have each successfully increased coverage, usage and equity of distribution among the intervention areas [7], [8], [10], [11], [21], [35], [36]. Despite the dramatic success of these campaigns to increase both ownership and usage of ITNs, the proportion of ITN users remains less than that of ITN owners. Recent data show that despite the proportion of households that own an ITN increased to 65% in one study, the use of ITNs among currently pregnant women the night before the survey in that study was only 23% [10].

In addition to the increases in ownership and use seen in intervention areas, survey data from African countries have reported up to a doubling of ITN ownership and use among pregnant women between survey periods of 3–4 years, which may be attributable to increases in national ITN promotional activities such as: reducing or eliminating taxes and tariffs; creating demand; making nets affordable through subsidies or vouchers; free mass distribution programs, and/or stimulating the commercial market [37]–[39]. However, the gap between the proportion of pregnant women sleeping under an ITN and the proportion of households owning an ITN persists.

Apart from the studies described above evaluating the effect of distribution strategies or national efforts to increase ITN coverage and use, additional studies have assessed factors associated with bed net ownership or use during pregnancy. One study examined correlates of bed net ownership during pregnancy [21]. This was a small study (n = 351) of women attending ANC in the Congo who were given ITNs during enrollment. Prior to enrollment, prevalence and predictors of ownership were assessed. Later, correlates of bed net usage the night before delivery were examined [22]. Two other studies looked at factors associated with bed net usage during pregnancy collectively among owners and non-owners of bed nets. The first was a community-based survey of 976 women who had been pregnant in the last year in Kenya [18]. The definition of ITN usage during pregnancy was not described in this study. The other study was conducted among a cohort of post-natal women (n = 293) delivering in a hospital without complications in Tanzania [20]. In this study, ITN usage was defined as having used an ITN for more than 75% of the pregnancy. However, in this study only a small subset of the variables collected were presented in relation to ITN usage.

Socio-demographic factors associated with net use during pregnancy have been evaluated in few studies. In one study, women 30 years or older were nearly 4 times more likely to use a net compared to women less than 20 years (OR: 3.8; 95% CI: 1.3, 5.0), and women 20–29 years were 2.5 times more likely to own a net compared to women less than 20 years (OR: 2.5; 95% CI: 1.5, 9.4) [20]. However, age was unrelated to net usage in a second study and in ours [22]. Neither education nor marital status were associated with net use in our study or in two earlier ones [18], [22]. Parity was investigated as a correlate of net usage in two studies. Primiparous women were less likely to use a net compared to multiparious women (RR: 0.46; 95% CI: 0.22, 0.97) in one study [22], whereas parity was not associated with net usage in the other study [18] or in ours. Knowledge of malaria was investigated in one study: a high score was associated with more than a two-fold increased likelihood of net usage compared to a low score [20]. However, in our study knowledge of malaria score was not related to of bed net usage.

It has been reported that the cost of ITNs and their relative non-availability for purchase at local vendors are a major deterrent of ITN ownership and subsequent use across Africa [7], [19], [21], [23], [40], [41]. Indicators of wealth status have been investigated as correlates of net usage during pregnancy in 2 studies [18], [20], with conflicting results. Having a lower wealth status was associated with use of a bed net during pregnancy (OR: 2.5; 95% CI: 1.4, 4.7) in one study [18], whereas ownership of a radio (suggestive of a higher wealth status) was associated with net using in another study (OR: 2.3; 95% CI: 1.0, 5.5) [20]. In the current study among households in which a net was present, women in the poorest households were more likely to use a net during pregnancy than those in households that were less poor. Owning more than 1 net was associated with nearly a two-fold increased likelihood of using a net in pregnancy (OR: 1.9; 95% CI: 1.0, 3.8) [22]. In our study, women whose household owned two or more nets during pregnancy were slightly more likely to always use a net compared to women who owned 1 net.

The results of our study are subject to the following limitations. It is possible that the insecticide on some of ITNs referenced in this study was no longer active during the exposure period. This would result in an overestimate of the prevalence of effective ITN use during pregnancy. The standard question many people use to define a net as insecticide-treated is to ask if the net has been re-treated within the last 12 months. However, a study in Tanzania found that people wash their net on average 4–7 times per year with soap, and that 67% of the nets reported to have been re-treated in the last 12 months had an insufficient amount of insecticide (<5mg/m^2^) [42]. We could not identify a series of questions which could be asked quickly and reliably to provide an accurate assessment of ITN status during the most recent pregnancy, and therefore the type of net was the best proxy in this setting. All but 1 woman reported the net they slept under most often during their pregnancy was either a net pretreated with insecticide, or a long lasting ITN. Use of a WHO-recognized long lasting ITN (PermaNet, Olyset, or Interceptor) was reported by 46% of women who owned a bed net. An additional 5% of women used a KO net, which is marketed as a long lasting ITN [43]. While the remaining 49% of the nets were identified as pretreated ITNs, we did not ask women how old the ITN was at the time of their pregnancy, or when it was last re-treated. Additionally, because our outcome measures were based on self-report, social desirability bias has the potential to create differential misclassification of the outcome resulting in a spuriously high estimate. For example, women who know ITN interventions are desirable and recommended may be more likely to report always using them when they did not. We did not ascertain where women obtained their net from and therefore were limited to explore the extent to which free net distribution campaigns increased usage in this population. Furthermore, recall bias could have resulted in non-differential misclassification biasing the results towards the null. Lastly, our inclusion criteria excluded women with a pregnancy loss before the third trimester of pregnancy; this group may have different determinants of ITN ownership and use compared to our population.

Several aspects of the methodology used in the study helped to strengthen the results. This was a population-based random sample of recently pregnant women. The use of several visual aids sought to reduce recall bias throughout the interview, including the creation of a pregnancy history calendar in which each pregnancy was mapped out over time. ITN usage during pregnancy was separated into trimesters, and women were shown the trimesters on the calendar and then asked to report their use of ITNs during these three periods separately. Breaking the pregnancy into trimesters and assessing net usage by trimester can better measure the variability of bed net usage that may occur with changes in the seasons, and possible changes in a woman's risk perception as her pregnancy progresses. Because we were retrospectively interviewing women regarding their behaviors during their last pregnancy, we were unable to utilize the standard single question, “Did you sleep under a bed net last night”. However, we believe that asking women about using separately during each trimester may provide a better assessment of ITN usage during pregnancy than a single question. Visual aids were used to improve communication between the interviewer and respondent. A comprehensive conceptual framework was developed which served as the basis for the questionnaire, and a quantitative approach was used to analyze the data.

After all interviews were completed, we returned to the homes of participants and installed a rectangular shaped long-lasting ITN. We attempted to maximize convenience of the bed nets by utilizing multiple attachment points, and by showing the women how to tie the net up during the day to increase space. We assumed that by supplying the nails and string, and installing the nets ourselves, we could increase the likelihood that they would be used. By providing and installing the nets for the participants, it was clear how challenging daily ITN use is in this setting. The houses are typically small, crowded, and often lack proper ventilation. In our interview, we attempted to ask an open question on what could make it easier to sleep under an ITN more often. However, the participants found this question difficult to answer and it was eventually removed from the questionnaire. Among the responses that we did receive, a third of participants mentioned either increasing the size of their home, or improving ventilation would make it easier to use the nets more regularly.

In summary, we evaluated correlates ITN usage during pregnancy among ITN owners. While there were some modest associations seen, we were unable to pinpoint why women did not adhere to the WHO recommendations of always using an ITN during pregnancy. Until better predictors of use are identified, it is not clear what measures might succeed in increasing ITN usage. However, our experience of installing nets in this setting suggests that household-level challenges to the installation of ITNs – such as the ratio of sleeping room size to ITN size and lack of appropriate fixture points to hang ITNs – may be important unmeasured influences on ITN usage. We would therefore recommend that more data are needed on the capacity of individual households to support the installation of ITNs. These types of data may provide insight into interventions that could improve the convenience and adherence of daily net use.
